# Multiple Fusion Based on the CCD and MEMS Accelerometer for the Low-Cost Multi-Loop Optoelectronic System Control

**DOI:** 10.3390/s18072153

**Published:** 2018-07-04

**Authors:** Yong Luo, Yao Mao, Wei Ren, Yongmei Huang, Chao Deng, Xi Zhou

**Affiliations:** 1 Institute of Optics and Electronics, Chinese Academy of Science, Chengdu 610209, China; ly250047087@126.com (Y.L.); maoyao@ioe.ac.cn (Y.M.); renwei9327@163.com (W.R.); chaosir1991@gmail.com (C.D.); zhouxiee@mail.ustc.edu.cn (X.Z.); 2 Key Laboratory of Optical Engineering, Chinese Academy of Sciences, Chengdu 610209, China; 3 University of Chinese Academy of Sciences, Beijing 100039, China

**Keywords:** multiple fusion, the optoelectronic control system, disturbance suppression, low-cost, disturbance observer, virtual velocity loop

## Abstract

In the charge-coupled device (CCD) and micro-electro-mechanical system (MEMS) accelerometer based low-cost multi-loop optoelectronic control system (OCS), due to accelerometers’ drift and noise in low frequency, the disturbance suppression (DS) is insufficient. Previously, based on the acceleration and position dual-loop control (ADLC), researchers combined a disturbance observer (DOB) with a virtual velocity loop to make some medium-frequency DS exchange for low-frequency performance. However, it is not optimal because the classic DOB based on accelerometers’ inaccurate signals cannot observe accurate disturbance in low frequency and the velocity based on a CCD and accelerometer time-domain fusion carried the CCD’s delay, resulting in the drop of medium-frequency DS. In this paper, considering the CCD’s advantage in low frequency and the accelerometer’s strength in high frequency, we propose to fuse their signals twice with a modified complementary filter method to respectively acquire an acceleration and velocity. The new acceleration with no drift and less noise but lower bandwidth creates a new acceleration model and is only used in fusion DOB (FDOB), while the velocity with little delay is to build an additional velocity loop. Compared with the traditional DOB enhanced by the time-domain fusion velocity loop, experiments verify that the proposed multiple fusion would apparently enhance the system’s DS, especially in low and medium frequency.

## 1. Introduction

The CCD-based optoelectronic control system (OCS) is an important component in the high-precision capture and tracking optoelectronic platform, which is commonly used in astronomical observation, laser communication, target tracking and line-of-sight stabilization [1,2,3,4]. With the expansion of generalization and commercialization, the OCS becomes smaller and is more installed on moving platforms, such as vehicles, airplanes, satellites, which are susceptible to external disturbance. Limited by the CCD’s delay and low-sampling rate, the disturbance suppression(DS) performance of the outer position loop is extremely restricted [5,6]. Generally, to enhance the DS, we attempted to use inertial sensors to build a high-sampling rate inner loop [7,8]. As a member of the inertial sensors family, the MEMS accelerometer has advantages with low price, small size, high precision and high bandwidth and has been commonly used in telescopes, navigation and robots [9,10,11,12]. However, the MEMS accelerometer also has a defect that in low frequency its signal is commonly polluted by noise and drift, leading to an insufficient DS in low frequency. Therefore, many researchers used a group of the accelerometer and gyro to guarantee a good DS [11,13]. But more sensors mean more cost and space that is not suitable for the small and cost-efficient OCS. Previously, in the MEMS accelerometer and CCD based OCS, based on the acceleration and position dual-loop control(ADLC), Reference [14] combined an acceleration disturbance observer(DOB) with an additional virtual velocity loop to make some medium-frequency DS exchange for low-frequency performance, barely getting a better DS. Nevertheless, it is not optimal because the classic DOB based on accelerometers’ inaccurate signals cannot observe accurate disturbance in low frequency and the velocity based on the time-domain fusion of a CCD and accelerometer carries the CCD’s delay, resulting in a big drop of medium-frequency DS. To solve the problem, the most important is to get a more precise acceleration and velocity based on the existing sensors.

As we know, when the OCS works to stabilize the line-of-sight, the CCD’s data represent the platform’s position signal. Although the CCD’s signal is accompanied with imaging delay, its low-frequency component is still very good because the low-frequency signal’s phase lag caused by the imaging delay is very small. On the contrary, the MEMS accelerometer with a high bandwidth has strength in high frequency while in low frequency its signal is weak and susceptible to drift and noise. Therefore, it is natural to combine the CCD’s low-frequency signal with the MEMS accelerometer’s relative high-frequency signal to get a better fusion velocity and acceleration. Sensors fusion has always been a hot topic. The common methods include the complementary filter way, the gradient descent algorithm and the Kalman way and so forth [15,16,17]. Gradient descent method is easy to fall into a local optimum. The Kalman filter as a time-domain filter needs to build a stable state space equation in which the sensors’ noise and biases are often treated as a stochastic framework. And it has been successfully applied in some follow-up systems with high-sampling rate sensors free of delay [18,19]. However, due to the CCD’s non-negligible delay in this paper, the Kalman filter needs to predict the state of the future, which costs a lot of calculation and puts high demands on the hardware configuration. The complementary filter way proposed in this paper is mainly accomplished by a pair of complementary filters, which can respectively extract low-frequency and high-frequency signals. Compared with the other methods, the complementary filter based on frequency-domain fusion is easy to implement in engineering and can output a high-bandwidth motion state, without a large computational effort. Moreover, with a spectrum fitting method in system identification, we can directly deduce the platform’s transfer function, which is very suitable for the frequency-domain analysis and model reference control.

In this paper, we propose to use the complementary filter method to twice fuse the signals of the CCD and MEMS accelerometer, respectively getting a fusion acceleration and velocity without additional sensors. Based on the original ADLC structure, the new acceleration with no drift and less noise, would be used in fusion DOB(FDOB), while the velocity nearly with no delay is to build an additional velocity loop. Since the DOB method is often used to compensate the low and medium disturbance and the MEMS accelerometer’s high-frequency noise would also affect the accuracy of observed disturbance, we modified the traditional complementary filter method and only combined the MEMS accelerometer’s medium-frequency signal with the CCD’s low-frequency signal to rebuild the acceleration model and get a relatively low-bandwidth acceleration. To avoid the low-bandwidth acceleration decreasing the system’s bandwidth, the acceleration feedback loop would still use the MEMS accelerometer’s original signal. Series of analysis and experiments verified that the virtual sensors have very high precision and the proposed way would apparently enhance the system’s DS, especially in low and medium frequency.

This paper is organized as follows. Section 2 respectively descripts the previous enhanced-DOB(EDOB) method and the novel FDOB method and gives out the basic theory of the complementary filter. Section 3 focuses on the added virtual velocity loop and the system’s robustness. Section 4 discusses the modified complementary filter and the details of the fusion acceleration and velocity. Section 5 is the experiment part which respectively presents the DS improvement from the FDOB and the virtual velocity loop. Concluding remarks are presented in Section 6.

## 2. EDOB and FDOB

### 2.1. The EDOB Built in the Acceleration Control Loop

**Figure 1:**
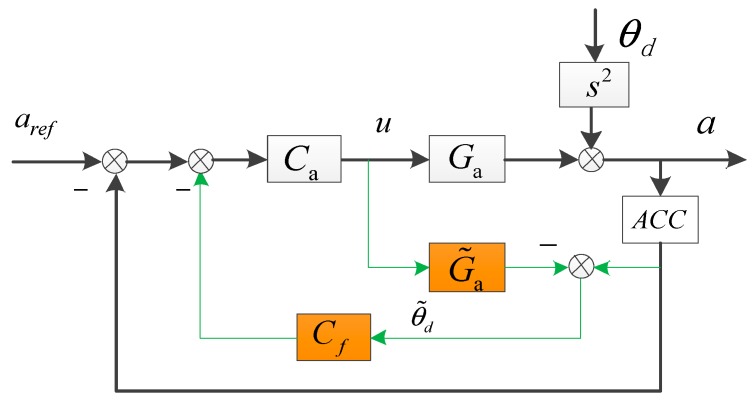
The EDOB method. $G_{a}$ is the acceleration open-loop transfer function. ${\tilde{G}}_{a}$ is the approximate model of the platform. $C_{a}$ and $C_{f}$ is respectively the acceleration controller and the controller of disturbance compensation. ACC respects the MEMS accelerometer. $a_{ref}$, a is the given acceleration and the output acceleration. $\theta_{d}$ is the external disturbance.

DOB as a disturbance compensation feed forward method, has commonly been used in industrial control [20,21]. As it does not need to add an additional sensor and basically does not affect the stability of the system, it is very suitable for the small OCS whose model can be recognized by spectrum fitting and seldom changes in motion. Deng first introduced the DOB disturbance feed forward control into the OCS and later proposed a modified method called EDOB [13,22]. Figure 1 presents the structure of EDOB. Compared with the DOB, EDOB changed the compensation object $G_{a}$ to be the open-loop transfer function $C_{a}G_{a}$ and optimized the controller design.

The closed-loop acceleration is given as follows.

(1) $$a=uG_{a}+\theta_{d}S^{2}$$

(2) $$u=\left[a_{ref}-a-(a-u{\tilde{G}}_{a})C_{f}\right]C_{a}$$

As u is a substitutable factor, after substitution, we get

(3) $$a=\frac{C_{a}G_{a}}{1+C_{a}G_{a}+C_{f}C_{a}(G_{a}-{\tilde{G}}_{a})}a_{ref}+\frac{(1-C_{f}C_{a}{\tilde{G}}_{a})s^{2}}{1+C_{a}G_{a}+C_{f}C_{a}(G_{a}-{\tilde{G}}_{a})}\theta_{d}$$

Since we only focus on the line-of-sight stabilization, $a_{ref}$ could be set as 0. The acceleration loop’s DS transfer function is as Equation (4).

(4) $$E_{EDOB}=\frac{a}{\theta_{d}s^{2}}=\frac{1-C_{f}C_{a}{\tilde{G}}_{a}}{1+C_{a}G_{a}+C_{f}C_{a}(G_{a}-{\tilde{G}}_{a})}$$

Theoretically, in Equation (4), if $C_{f}$ is set to be ${\left(C_{a}{\tilde{G}}_{a}\right)}^{-1}$, $E_{EDOB}$ would become 0. However, due to model mismatch and noise effects, the DS improvement is limited, especially in low frequency. The reason is that the observed disturbance in low frequency from the MEMS accelerometer is not accurate with a big drift and much noise and would lead to poor effects of compensation. In order to get a satisfying low-frequency DS, we first need to acquire a relatively accurate acceleration signal depending on the existing sensors, decreasing the influence of the drift and noise.

### 2.2. The FDOB Control

**Figure 2:**
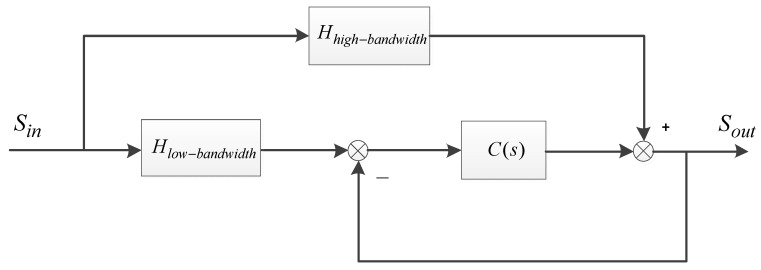
The basic principle of the complementary filter. $H_{high-bandwidth}$ and $H_{low-bandwidth}$ are respectively the transfer characteristics of the high-bandwidth and low-bandwidth sensors. C(s) is a designed open-loop filter. $S_{in}$ and $S_{out}$ are respectively the real motion status and the fusion output.

In OCS, the CCD’s low-frequency signal is very valuable. On the contrary, although the accelerometer has a bad low-frequency signal, it has a high bandwidth (around 1000 Hz) nearly with no delay. It is reasonable to combine the CCD with the MEMS accelerometer to get a fusion acceleration. The basic principle of the proposed complementary filter is shown in Figure 2.

From Figure 2, we get

(5) $$\begin{array}{cc} S_{out}\; & =\;[\frac{C(s)}{1+C(s)}H_{low-bandwidth}+\frac{1}{1+C(s)}H_{high-bandwidth}]\cdot S_{in}\\ & =\;[B_{closed}\cdot H_{low-bandwidth}+B_{restrain}\cdot H_{high-bandwidth}]\cdot S_{in} \end{array}$$

The closed-loop transfer function $B_{closed}$ is a low-pass filter which is to extract the low-bandwidth sensor’s signal, while the restrain transfer function $B_{restrain}$ is a high-pass one to filter the high-bandwidth sensor’s signal. If $H_{low-bandwidth}=H_{high-bandwidth}$, the fusion output is equal to the real motion status. However, even if $H_{low-bandwidth}\neq H_{high-bandwidth}$, it does not matter because we can also calculate the real model with spectrum fitting. This is equivalent to rebuilding the platform’s model and producing a virtual sensor.

**Figure 3:**
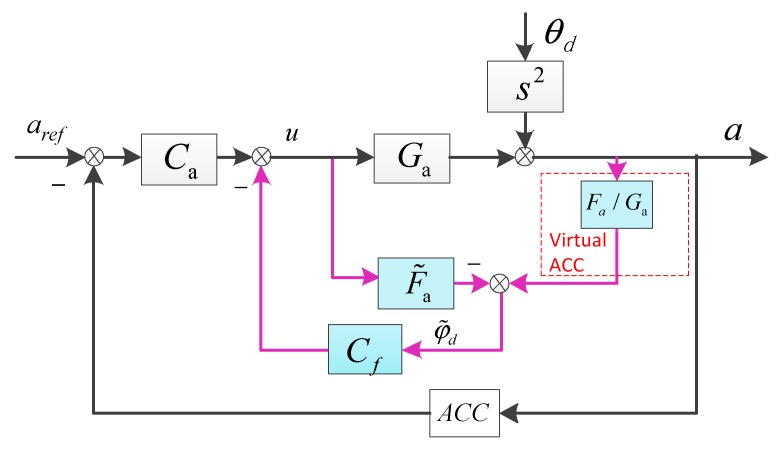
The FDOB method.

Suppose the platform’s new acceleration model is $F_{a}(s)$ under the complementary filter method, $F_{a}(s)/G_{a}(s)$ represents the transfer characteristic of the virtual accelerometer. Depending on $F_{a}(s)$, we build a new DOB structure called FDOB, which is shown in Figure 3.

The closed-loop acceleration is given as follows

(6) $$a=uG_{a}+\theta_{d}S^{2}$$

(7) $$u=(a_{ref}-a)C_{a}-(a\frac{F_{a}}{G_{a}}-u{\tilde{F}}_{a})C_{f}$$

After substitution,

(8) $$a-\theta_{d}S^{2}=(a_{ref}-a)C_{a}G_{a}-[aF_{a}-(a-\theta_{d}S^{2}){\tilde{F}}_{a}]C_{f}$$

So,

(9) $$a=\frac{C_{a}G_{a}}{1+C_{a}G_{a}+C_{f}(F_{a}-{\tilde{F}}_{a})}a_{ref}+\frac{(1-C_{f}{\tilde{F}}_{a})s^{2}}{1+C_{a}G_{a}+C_{f}(F_{a}-{\tilde{F}}_{a})}\theta_{d}$$

Similarly, we set $a_{ref}=0$, the acceleration loop DS of the FDOB method is as follows.

(10) $$E_{FDOB}=\frac{a}{\theta_{d}s^{2}}=\frac{1-C_{f}{\tilde{F}}_{a}}{1+C_{a}G_{a}+C_{f}(F_{a}-{\tilde{F}}_{a})}$$

Compared with Equation (4), the compensation object has changed to be ${\tilde{F}}_{a}(s)$. Different from directly using the accelerometer’s data, the fusion acceleration is better with no drift and less noise in low frequency, which means we can observe a more accurate outer disturbance and the low-frequency DS would be much more improved than by the EDOB method. 

Unfortunately, the FDOB as a feedforward way would not solve all the problems. Due to the existing of residual noise, the effect of compensation would be poor and even terrible in lower frequency, which is faced by all the feedforward methods. Considering that a feedback loop could get a converged result and naturally suppress noise’s influence, we attempt to build a virtual velocity loop between the acceleration loop and the position loop.

## 3. The Fusion Virtual Velocity Loop

**Figure 4:**
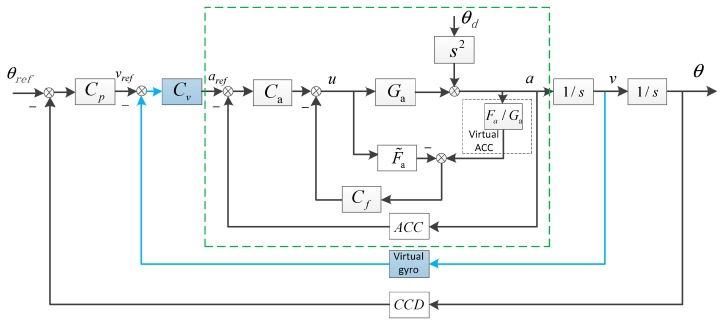
The TLC structure with FDOB. $C_{v}$ and $C_{p}$ are respectively the velocity controller and position controller. $\theta_{ref}$ respects the given position. θ is the output position.

After a virtual velocity loop is added, the ADLC system turns to be a triple-loop control(TLC) structure which is exhibited in Figure 4. The fusion method in Reference [14] is based on time domain, which would bring the CCD’s time delay to the velocity no matter in low frequency or high frequency and caused a big drop of DS in relatively high frequency. The proposed complementary filter method is based on frequency domain. Since we combine the low-frequency signal of the CCD which is less affected by the delay with the high-frequency signal of the MEMS accelerometer, we minimize the impact of delay on the system.

The whole DS without and with a virtual velocity loop is respectively as follows.

(11) $$\begin{array}{cc} E_{ADLC-FDOB} & =\frac{\theta }{\theta_{d}}=\frac{1-C_{f}{\tilde{F}}_{a}}{1+C_{a}G_{a}+C_{p}C_{a}G_{a}\frac{1}{s^{2}}+(F_{a}-{\tilde{F}}_{a})C_{f}}\;\approx \frac{1-C_{f}{\tilde{F}}_{a}}{1+C_{a}G_{a}+C_{p}C_{a}G_{a}\frac{1}{s^{2}}}\\ & =\frac{1}{1+C_{a}G_{a}}\cdot \frac{1-C_{f}{\tilde{F}}_{a}}{1+C_{p}\frac{C_{a}G_{a}}{1+C_{a}G_{a}}\frac{1}{s^{2}}}\approx \frac{1}{1+C_{a}G_{a}}\cdot \frac{1-C_{f}{\tilde{F}}_{a}}{1+C_{p}\frac{1}{s^{2}}} \end{array}$$

Similarly,

(12) $$\begin{array}{cc} E_{TLC-FDOB} & =\frac{\theta }{\theta_{d}}=\frac{1-C_{f}{\tilde{F}}_{a}}{1+C_{a}G_{a}+C_{v}C_{a}G_{a}\frac{1}{s}+C_{p}C_{v}C_{a}G_{a}\frac{1}{s^{2}}+C_{f}(F_{a}-{\tilde{F}}_{a})}\\ & \approx \frac{1-C_{f}{\tilde{F}}_{a}}{1+C_{a}G_{a}+C_{v}C_{a}G_{a}\frac{1}{s}+C_{p}C_{v}C_{a}G_{a}\frac{1}{s^{2}}}\\ & =\frac{1}{1{+C}_{a}G_{a}}\cdot \frac{1}{1+\frac{1}{s}\frac{C_{a}G_{a}}{1{+C}_{a}G_{a}}C_{v}}\cdot \frac{1-C_{f}{\tilde{F}}_{a}}{1+\frac{1}{s}\frac{\frac{1}{s}\frac{C_{a}G_{a}}{1{+C}_{a}G_{a}}C_{v}}{1+\frac{1}{s}\frac{C_{a}G_{a}}{1{+C}_{a}G_{a}}C_{v}}C_{p}}\\ & \approx \frac{1}{1{+C}_{a}G_{a}}\cdot \frac{1}{1+C_{v}\frac{1}{s}}\cdot \frac{1-C_{f}{\tilde{F}}_{a}}{1+C_{p}\frac{1}{s}} \end{array}$$

Generally, we choose to design the open-loop transfer function to be an integral element in order to get an adequate phase and amplitude margin. So if the controllers can be designed ideally, obviously, $\left|1/(1+C_{v}\cdot (1/s))\right|<1$. And $\left|E_{TLC-FDOB}\right|$ would be smaller than $\left|E_{ADLC-FDOB}\right|$, which means the whole DS would be enhanced in all frequency domain. 

As we know, the system’s robustness is a key indicator of the control performance. And it refers to the system’s sensitivity to parameter changes. Since the ADLC and TLC-FDOB both have a same position loop, we just need to examine the velocity sensitivity transfer function.

The sensitivity functions of ADLC and TLC-FDOB are as follows.

(13) $$S_{ADLC}=\frac{\left(\frac{C_{a}(G_{a}+\Delta G_{a})}{1+C_{a}(G_{a}+\Delta G_{a})}\cdot \frac{1}{s}-\frac{C_{a}G_{a}}{1+C_{a}G_{a}}\cdot \frac{1}{s}\right)/\left(\frac{C_{a}G_{a}}{1+C_{a}G_{a}}\cdot \frac{1}{s}\right)}{\Delta G_{a}/G_{a}}=\frac{1}{1+C_{a}(G_{a}+\Delta G_{a})}\approx \frac{1}{1+C_{a}G_{a}}$$

(14) $$\begin{array}{cc} S_{TLC-FDOB} & =\frac{({B^{\prime }}_{v}-B_{v})/B_{v}}{\Delta G_{a}/G_{a}}\\ & =\frac{1-{\tilde{F}}_{a}C_{f}}{1+C_{a}(G_{a}+\Delta G_{a})+(\frac{\Delta G_{a}+G_{a}}{G_{a}}\cdot F_{a}-{\tilde{F}}_{a})C_{f}+C_{v}C_{a}(G_{a}+\Delta G_{a})\cdot \frac{1}{s}}\\ & \approx \;\frac{1-{\tilde{F}}_{a}C_{f}}{1+C_{a}G_{a}+C_{v}C_{a}G_{a}\cdot \frac{1}{s}}\;=\;\frac{1}{1+C_{a}G_{a}}\cdot \frac{1-{\tilde{F}}_{a}C_{f}}{1+C_{v}\cdot \frac{C_{a}G_{a}}{1+C_{a}G_{a}}\cdot \frac{1}{s}}\\ & \approx \;\frac{1}{1+C_{a}G_{a}}\cdot \frac{1-{\tilde{F}}_{a}C_{f}}{1+C_{v}\cdot \frac{1}{s}} \end{array}$$

where

(15) $$B_{v}=\frac{C_{v}C_{a}G_{a}\cdot \frac{1}{s}}{1+C_{a}G_{a}+(F_{a}-{\tilde{F}}_{a})C_{f}+C_{v}C_{a}G_{a}\cdot \frac{1}{s}},\;{B^{\prime }}_{v}=\frac{C_{v}C_{a}(G_{a}+\Delta G_{a})\cdot \frac{1}{s}}{1+C_{a}(G_{a}+\Delta G_{a})+(\frac{\Delta G_{a}+G_{a}}{G_{a}}\cdot F_{a}-{\tilde{F}}_{a})C_{f}+C_{v}C_{a}(G_{a}+\Delta G_{a})\cdot \frac{1}{s}}$$

Apparently, according to the above analysis, $\left|S_{TLC-FDOB}\right|<\left|S_{ADLC}\right|$, which signifies that the FDOB and additional virtual velocity loop could also improve the robustness of the traditional ADLC structure. If the parameters of the controlled object change a little because of external interference, the system’s stability of the TLC-FDOB would suffer less than that of the ADLC.

## 4. The Complementary Filter Method and Performance Analysis

### 4.1. The Fusion Acceleration Based on the Modified Complementary Filter Method

According to Equation (5), considering the CCD’s delay $e^{-\tau s}$, we can get the transfer function of the virtual accelerometer as follows. $H_{CCD}$ respects the transfer characteristic of the CCD without delay, $H_{ACC}$ is the transfer characteristic of the MEMS accelerometer.

(16) $$\begin{array}{cc} \frac{a_{fusion}}{a_{real}} & =\;\frac{C(s)}{1+C(s)}\cdot s^{2}e^{-\tau s}H_{CCD}+\frac{1}{1+C(s)}H_{ACC}\\ & =\;B_{closed}\cdot s^{2}e^{-\tau s}H_{CCD}+B_{restrain}\cdot H_{ACC}\\ & =\;\frac{F_{a}(s)}{G_{a}(s)}\; \end{array}$$

When in very low frequency, $B_{restrain}$ approaches to 0, $a_{fusion}$ is equal to the acceleration got through the CCD with no drift. Similarly, when in relatively high frequency, $B_{closed-loop}$ approaches to 0, the $a_{fusion}$ is equal to the acceleration got by the MEMS accelerometers with nearly no delay. In medium frequency, $a_{fusion}$ contains both the CCD’s and MEMS accelerometers’ signals. 

However, in fact, although the original complementary filter method can eliminate the low-frequency drift, it still cannot get a very satisfied acceleration because of the MEMS accelerometer’s high-frequency noise. 

Assume $R_{CCD}$ and $R_{ACC}$ are respectively the CCD’s signal without delay and the MEMS accelerometer’s signal and they contain both useful signal and interference signal. According to Equation (16), we can deduce the fusion acceleration as follows.

(17) $$\begin{array}{cc} a_{fusion}(s) & =\;C_{low-pass}(s)s^{2}e^{-\tau s}R_{CCD}+C_{high-pass}(s)R_{ACC}\\ & =\;C_{low-pass}(s)s^{2}e^{-\tau s}R_{CCD}+[1-C_{low-pass}(s)]R_{ACC}\\ & =\;R_{ACC}+C_{low-pass}(s)[s^{2}e^{-\tau s}R_{CCD}-R_{ACC}] \end{array}$$

The new fusion acceleration contains two items, the $R_{ACC}$ and the low-frequency data filtered by $C_{low-pass}$. When given a low-frequency input, *R_ACC_* not only contains a zero drift but also contains high-frequency noise. The second item of Equation (17) could help eliminate the drift of *R_ACC_* but cannot deal with the high-frequency noise signal, which will decrease the accuracy of the last acceleration. Therefore, in this case the basic complementary filter cannot work well. In order to prevent the pollution of the high-frequency noise, we change $C_{high-pass}$ to be a band-pass filter $C_{band-pass}$.

Considering that the high-order low-pass filter would bring a large phase lag, we choose a first-order low-pass filter as $C_{low-pass}$. Equation (18) presents the new fusion acceleration.

(18) $$\begin{array}{cc} a_{fusion}(s) & =C_{low-pass}(s)R_{CCD}s^{2}e^{-\tau s}+C_{band-pass}(s)R_{ACC}\\ & =\;\frac{1}{1+T_{1}s}R_{CCD}s^{2}e^{-\tau s}+\frac{T_{1}s}{1+T_{1}s}\frac{1}{1+T_{2}s}R_{ACC}\\ & \left(T_{2}<<\tau <<T_{1}\right) \end{array}$$

In Equation (18), although the double differential of $R_{CCD}$ to get the acceleration would amplify the impact of the noise, since the signal of noise’s differential mainly distribute on high-frequency domain, $C_{low-pass}$ will nearly eliminate the effect of the noise. Similarly, the band-pass filter $C_{band-pass}$ could cut down the low-frequency drift and decrease the pollution of the high-frequency noise. Hence, now we could ignore the drift and noise and get $R_{CCD}s^{2}\approx R_{ACC}\approx a_{real}$, then

(19) $$a_{fusion}(s)\;\approx (\frac{1}{1+T_{1}s}e^{-\tau s}+\frac{T_{1}s}{1+T_{1}s}\frac{1}{1+T_{2}s})a_{real}\;=\frac{T_{1}s+(1+T_{2}s)e^{-\tau s}}{\left(1+T_{1}s)(1+T_{2}s\right)}a_{real}\;$$

**Figure 5:**
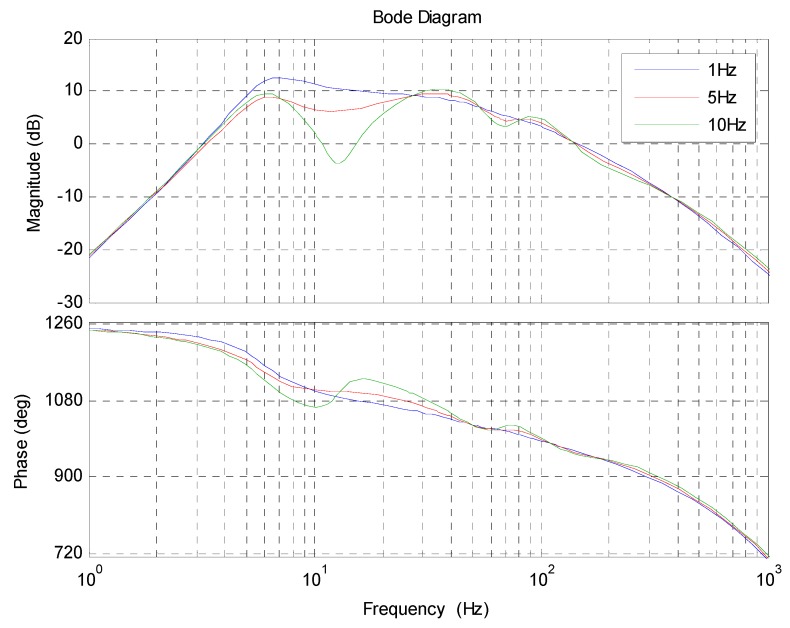
The simulation of the acceleration bode response with different $1/T_{1}$.

In Equation (19), the form of the acceleration transfer function has been fixed. The rest is to determine the handover frequency $1/T_{1}$. Theoretically, whatever the value of $1/T_{1}$ is, the final acceleration model could be identified by spectrum fitting. Nevertheless, in fact, $1/T_{1}$ will affect the linearity of the object, which will determine the difficulty of the controller design. Figure 5 exhibits the simulation of the acceleration bode response with different $1/T_{1}$.

In Figure 5, as $1/T_{1}$ increases, the curve fluctuates more and more violently. It is because the smaller handover frequency means less influence of the non-linear link $e^{-\tau s}$ on the fusion acceleration. Since the curve fluctuations are detrimental to controller design, we should try to choose a small handover frequency. However, since the drift and noise of the MEMS accelerometer have a serious pollution on the useful signal below 2 Hz, to reduce the MEMS accelerometer’s proportion in the fusion acceleration, $1/T_{1}$ should be no less than 2 Hz. Therefore, we choose 2 Hz as the handover frequency.

Since the non-linear link $e^{-\tau s}$ is not convenient for spectrum analysis, we give out its approximate form.

(20) $$e^{-\tau s}=\frac{1}{e^{\tau s}}=\frac{1}{1+\tau s+1/2\tau^{2}s^{2}+1/6\tau^{3}s^{3}\ldots }\approx \frac{1}{1+\tau s}\;(∵\tau =0.02<<1\;)$$

Substituting Equation (20) into Equation (19)

(21) $$\begin{array}{cc} a_{fusion}(s) & \approx \;(\frac{1}{1+T_{1}s}\frac{1}{1+\tau s}+\frac{T_{1}s}{1+T_{1}s}\frac{1}{1+T_{2}s})a_{real}\;=\frac{T_{1}\tau s^{2}+(T_{1}+T_{2})s+1}{\left(1+T_{1}s)(1+\tau s)(1+T_{2}s\right)}a_{real}\\ & =\frac{T_{1}\tau s^{2}+(T_{1}+T_{2})s+1}{\left[T_{1}\tau s^{2}+(T_{1}+\tau )s+1](1+T_{2}s\right)}a_{real}\;\approx \frac{1}{\left(1+T_{2}s\right)}a_{real}\;(∵T_{2}<<\tau <<T_{1}) \end{array}$$

Equation (21) is the approximation of Equation (19). Intuitively, the rebuilt acceleration object $F_{a}$ is to add an inertial element behind the original one. However, in fact, purely adding an inertial element could only filter the high-frequency noise but the complementary filter method would simultaneously eliminate the time-domain drift. Considering that the proposed way would also lead to a decrease of the acceleration’s bandwidth, in order to avoid the decrease of the acceleration closed-loop bandwidth, in the acceleration feedback loop we still use the MEMS accelerometers’ original signal. Since an accurate model in high frequency is commonly unreliable, with DOB method we generally choose to compensate low- and medium- frequency disturbance. What’s more, as a feed forward method, DOB would not affect the closed-loop performance. Therefore, the relatively low-bandwidth fusion acceleration is very suitable to the DOB method.

### 4.2. The Fusion Velocity

Similarly, according to Equation (5), we can differentiate the CCD’s signal and integrate the accelerometer’s signal to get the fusion velocity as follows.

(22) $$\begin{array}{cc} \frac{v_{fusion}}{v_{real}} & =\;\frac{C_{filter}}{1+C_{filter}}\cdot se^{-\tau s}H_{CCD}+\frac{1}{1+C_{filter}}\cdot \frac{1}{s}H_{ACC}\\ & =\;B_{closed}\cdot se^{-\tau s}H_{CCD}+B_{restrain}\cdot \frac{1}{s}H_{ACC}\\ & =\;\frac{F_{v}(s)}{G_{v}(s)}\; \end{array}$$

In Equation (22), we still choose a one order filter to extract the signals of the CCD and the MEMS accelerometer. Since the fusion velocity is used in closed-loop control, we choose a high-pass filter not a band-pass to extract the MEMS accelerometer’s signal, or it will result in the decrease of closed-loop bandwidth. Substitute the filters to Equation (22) as follows.

(23) $$\frac{v_{fusion}}{v_{real}}\;=\;\frac{1}{1+T_{1}s}\cdot e^{-\tau s}sH_{CCD}+\frac{T_{1}s}{1+T_{1}s}\cdot \frac{1}{s}H_{ACC}$$

In Equation (23), we can treat $sH_{CCD}\approx \frac{1}{s}H_{ACC}\approx v_{real}$, then

(24) $$v_{fusion}\approx \;(\frac{1}{1+T_{1}s}\cdot e^{-\tau s}+\frac{T_{1}s}{1+T_{1}s})v_{real}$$

Substitute Equation (20) into Equation (24)

(25) $$\begin{array}{cc} v_{fusion} & =\;(\frac{1}{1+T_{1}s}\cdot e^{-\tau s}+\frac{T_{1}s}{1+T_{1}s})v_{real}\;\approx \;(\frac{1}{1+T_{1}s}\cdot \frac{1}{1+\tau s}+\frac{T_{1}s}{1+T_{1}s})v_{real}\\ & =\;\frac{T_{1}\tau s^{2}+T_{1}s+1}{T_{1}\tau s^{2}+(T_{1}+\tau )s+1}v_{real}\approx \;v_{real}\;(∵\tau <<T_{1})\; \end{array}$$

From Equation (25), the fusion velocity would have the same form with the platform’s real velocity but in fact, due to the influence of the delay, the natural frequency and damping coefficient may change slightly. The handover frequency $1/T_{1}$ could be 2 Hz, which is same with the fusion acceleration’s. With the spectrum fitting method, we can identify the detail parameters and give out the accurate transfer function.

## 5. Experimental Verification

**Figure 6:**
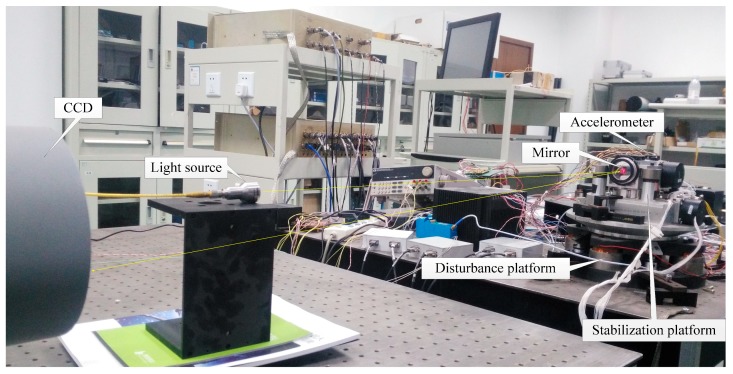
Experimental apparatus.

Figure 6 exhibits the experimental apparatus. As the OCS is a two-axis symmetrical system, we only take one axis into consideration. To simulate the external disturbance, an additional OCS platform is used as the pedestal which can be driven by the dynamic signal analyzer. The disturbance signal is a sine wave with variable frequency. The laser light source emits a light as a reference. The CCD receives the reflected light from the OCS to calculate the offset error to the center of the boresight. Two linear MEMS accelerometers (Model 1221, SILICON DESIGNS, Inc., Kirkland, WA, USA) work in a differential configuration to get the angle acceleration of one direction. The motion of the disturbance platform is measured by the eddy fixed on its bottom. The CCD works in a 100 Hz sampling rate with 0.02 s delay, while the MEMS accelerometers works in a 5000 Hz rate. In the processing of measuring the DS, the disturbance platform works on an open-loop mode, continuously outputting sine signal from 1~100 Hz. The stabilization platform works on a closed-loop mode to stabilize the visual axis. 

### 5.1. The FDOB Experiment Based on Acceleration and Position Dual-Loop Control

**Figure 7:**
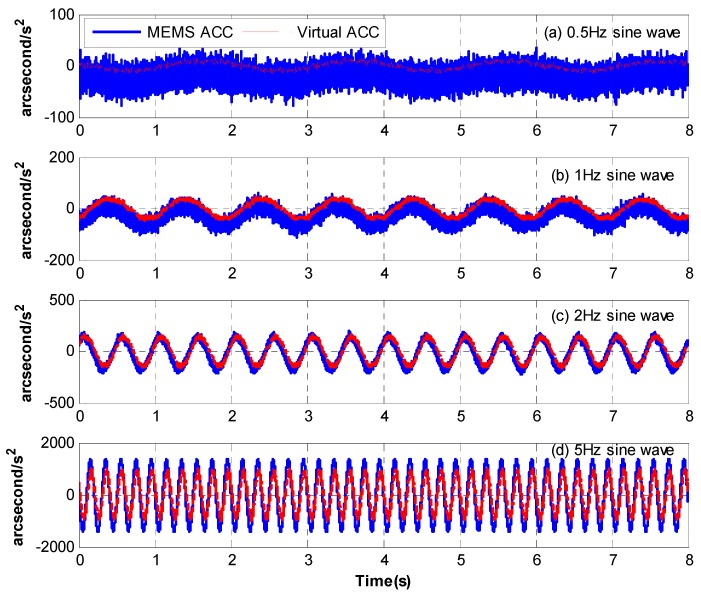
The time-domain curves of the MEMS accelerometer and the virtual accelerometer.

**Figure 8:**
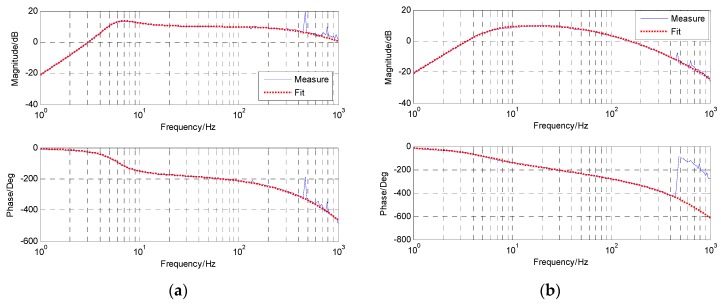
The open-loop bode response of acceleration. (**a**) ${\tilde{G}}_{a}$ based on the MEMS accelerometer; (**b**) ${\tilde{F}}_{a}$ based on the virtual accelerometer.

Figure 7 presents the time-domain curves of the MEMS accelerometer and the virtual accelerometer in different frequencies. Below 2 Hz, the MEMS accelerometer’s signal contains significant drift and noise, while the virtual accelerometer’s signal is much better with no drift and less noise, which means the modified complementary is valid. Above 2 Hz, the signals of the MEMS accelerometer and the virtual one are both good. Although they may have different amplitudes in the same frequency, it does not matter because we can identify the last transfer function with spectrum fitting. Figure 8 exhibits their open-loop bode responses from 1 Hz to 1 KHz. The blue line represents the response measured from the real platform, while the red line represents the fitting one from the math model. Obviously, the matching degree of fitting is very high. And the specific identified transfer functions are as follows.

(26) $${\tilde{G}}_{a}(s)=\frac{0.0022s^{2}}{0.007s^{2}+0.0185s+1}\cdot \frac{1}{0.0004s+1}$$

(27) $${\tilde{F}}_{a}(s)=\frac{0.0022s^{2}}{0.007s^{2}+0.04s+1}\cdot \frac{1}{0.0004s+1}\cdot \frac{1}{0.003s+1}$$

${\tilde{G}}_{a}(s)$ is the highly approximate model of the original acceleration model based on the MEMS accelerometer, which has a nearly 1 KHz bandwidth and is very suitable for closed-loop feedback control. ${\tilde{F}}_{a}(s)$ with no more than 100 Hz bandwidth, is the rebuilt acceleration model based on the virtual accelerometer, which is more suitable for DOB control. 

To measure the closed-loop DS of the OCS, firstly, we should design the acceleration controller $C_{a}$, which is usually designed as a lag controller.

(28) $$C_{a}=150\cdot \frac{0.0007s^{2}+0.0185s+1}{s(1+0.00077s)}$$

After the closed-loop acceleration has been designed, we can add the FDOB. According to Equation (12), $C_{f}$ should be designed as follows.

(29) $$C_{f-ideal}(s)=\frac{0.007s^{2}+0.04s+1}{0.0022s^{2}}\cdot (0.003s+1)\cdot (0.0004s+1)$$

In Equation (29), since the order of numerator is higher than the one of denominator, it cannot be accomplished in physics. The actually used controller is presented as Equation (30).

(30) $$\;C_{f}(s)=\frac{0.007s^{2}+0.04s+1}{0.0022s^{2}}\cdot \frac{\left(0.003s+1\right)}{\left(0.001s+1\right)}$$

From Equation (11), we know the improvement of DS is brought by the numerator, so we focus on its value after compensation.

(31) $$\begin{array}{l} 1-C_{f}{\tilde{F}}_{a}\\ =1-[\frac{0.007s^{2}+0.04s+1}{0.0022s^{2}}\cdot \frac{\left(0.003s+1\right)}{\left(0.001s+1\right)}]\cdot [\frac{0.0022s^{2}}{0.007s^{2}+0.04s+1}\cdot \frac{1}{0.0004s+1}\cdot \frac{1}{0.003s+1}]\\ =1-\frac{1}{\left(0.001s+1)(0.0004s+1\right)}=\frac{(4e-7)s^{2}+0.0014s}{\left(0.001s+1)(0.0004s+1\right)} \end{array}$$

**Figure 9:**
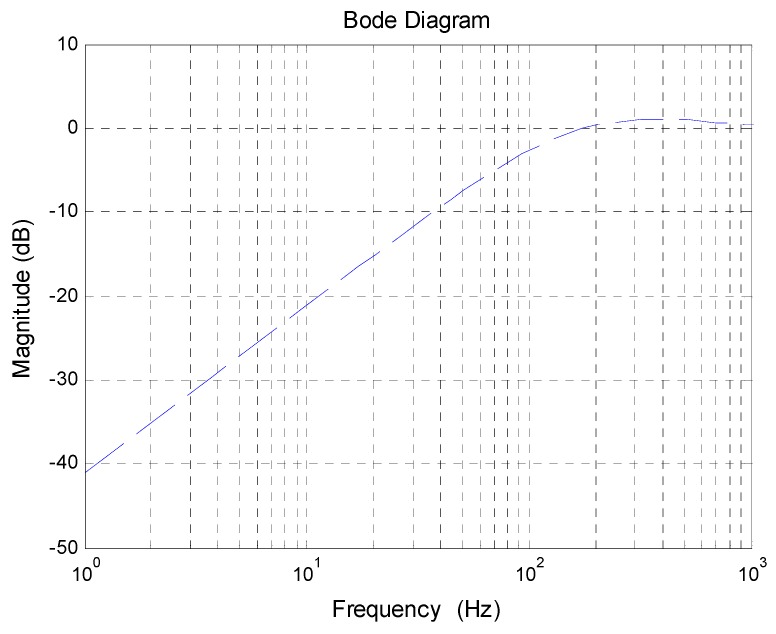
The simulation of DS improvement brought by FDOB.

Equation (31) is a high-pass filter as Figure 9. Compared with the traditional pure ADLC method, the low-frequency improvement is very apparent and as the frequency goes up, the promotion becomes smaller and smaller until there is no improvement, which is in line with forecasts.

**Figure 10:**
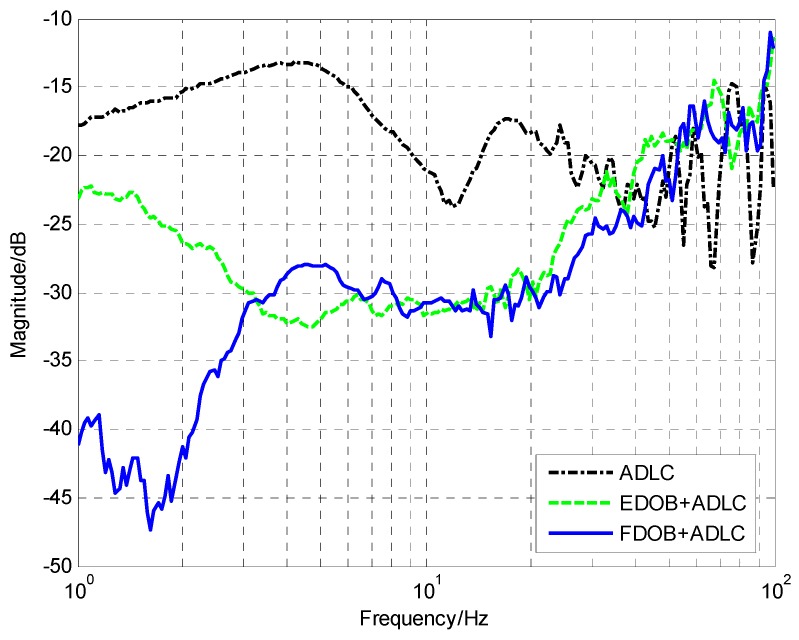
The improvement of DS brought by EDOB and FDOB.

Figure 10 presents the measured improvement of DS brought by EDOB and FDOB to the pure ADLC method. Obviously, the DS of the pure ADLC is not satisfied. Although the EDOB method could enhance the DS in low and medium frequencies, the low-frequency improvement is insufficient because of the MEMS accelerometer’s drift and much noise. Fortunately, the proposed FDOB has solved the problem. Compared with pure ADLC method, FDOB could extremely promote the low-frequency DSA and the maximum improvement could reach −30 dB in 1.8 Hz. Although the increase below 1.8 Hz has slowed down due to the effect of the residual noise, the improvement in low frequency is more apparent than in medium and high frequencies, which is coincident with the above simulation. In order to further promote the system’s DS and make full of sensors’ potential, we would continuously add the virtual velocity loop between the acceleration loop and the position loop.

### 5.2. The Virtual Velocity Loop with the FDOB

**Figure 11:**
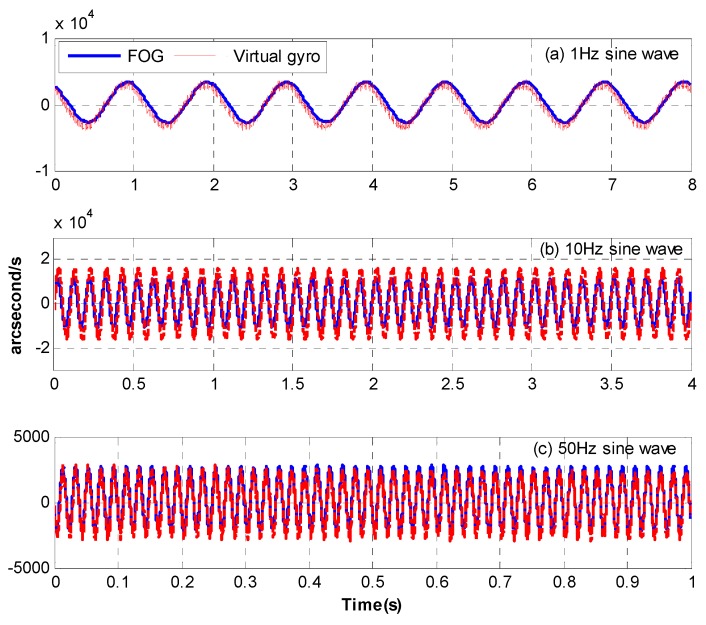
Time-domain wave of the FOG and virtual gyro. (**a**) 1 Hz sine wave, (**b**) 10 Hz sine wave, (**c**) 50 Hz sine wave.

**Figure 12:**
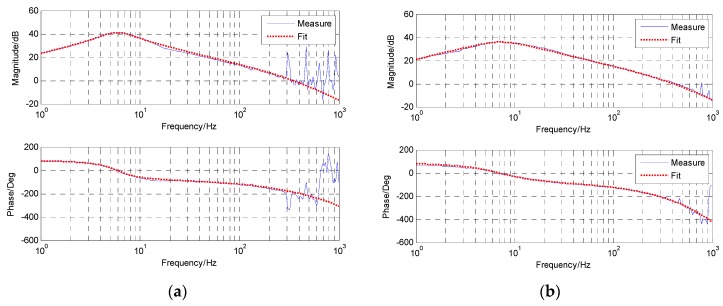
The open-loop bode responses of the velocity. (**a**) ${\tilde{G}}_{v}$ based on the FOG; (**b**) ${\tilde{F}}_{v}$ based on the virtual gyro.

Figure 11 presents the time-domain waves of the virtual gyro in different frequencies and we also present a group of signals of the fiber-optic gyro (FOG) (XW-FG70-20, Starneto, Beijing, China) as a comparison. The signal of the virtual gyro has a negligible phase lag and is very close to the real FOG’s. It means the frequency-domain fusion is much better than the time-fusion fusion in Reference [14], which would lead to the signal’s big phase lag at a relatively high frequency. Figure 12 shows the open-loop bode responses of the velocity from the FOG and virtual gyro. The identified transfer functions are as Equations (32) and (33). Their models are the same but the parameters are somewhat different.

(32) $${\tilde{G}}_{v}(s)=\frac{2.3s}{0.0072s^{2}+0.0202s+1}\cdot \frac{1}{0.0005s+1}$$

(33) $${\tilde{F}}_{v}(s)=\frac{1.8s}{0.000489s^{2}+0.02873s+1}\cdot \frac{1}{0.0004s+1}$$

From the open-loop transfer functions, although the resonant frequency and damping coefficient of the virtual gyro has changed, its bandwidth is very high and fits the closed-loop feedback control. Now, the ADLC feedback control has turned to be the TLC structure. $C_{a}$ and $C_{f}$ could be the same as the previous design. Since the inner acceleration loop has improved the platform’s characteristics, we can both use a low-pass filter as the velocity controller $C_{v}$ and the position controller $C_{p}$.

(34) $$C_{v}=0.15\cdot \frac{1}{1+0.00077s},\;C_{p}=12\cdot \frac{1}{1+0.0005s}$$

**Figure 13:**
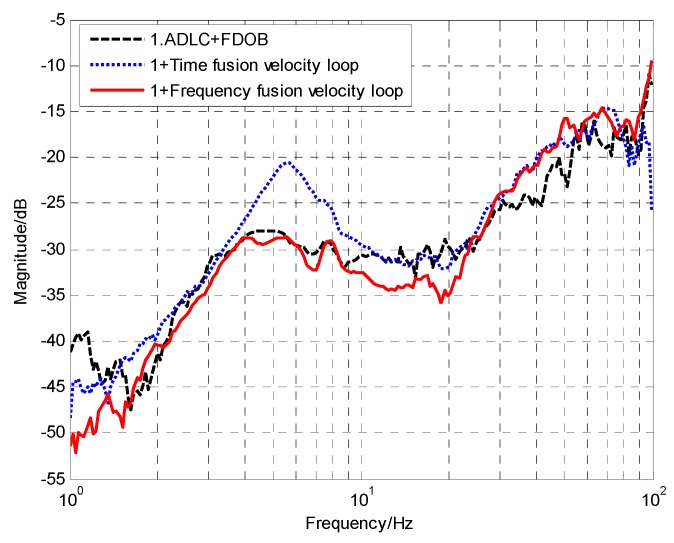
The comparison of DS with time-domain and frequency-main fusion methods.

Figure 13 shows the comparison of the DS with different fusion methods. Compared with the ADLC method enhanced by FDOB, the introduction of the virtual velocity loop with the time-domain fusion method in Reference [14] can enhance the low-frequency DS a little but the medium-frequency DS would decrease because of the CCD’s time delay. However, the virtual velocity loop based on the frequency-domain fusion could apparently improve the low- and medium- frequency DS. Although the virtual velocity loop cannot enhance the high-frequency DS, it does not matter because it commonly relies on the mechanical design. In total, after fusing the sensors’ data twice, the performance of the sensors has been fully utilized and we have obtained a satisfied system with extremely strong DS. 

**Figure 14:**
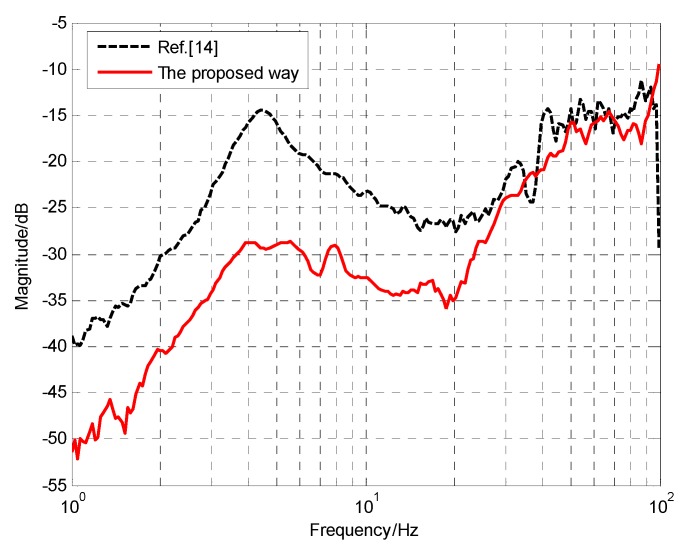
The DS’s comparison of Reference [14] and the proposed way.

**Figure 15:**
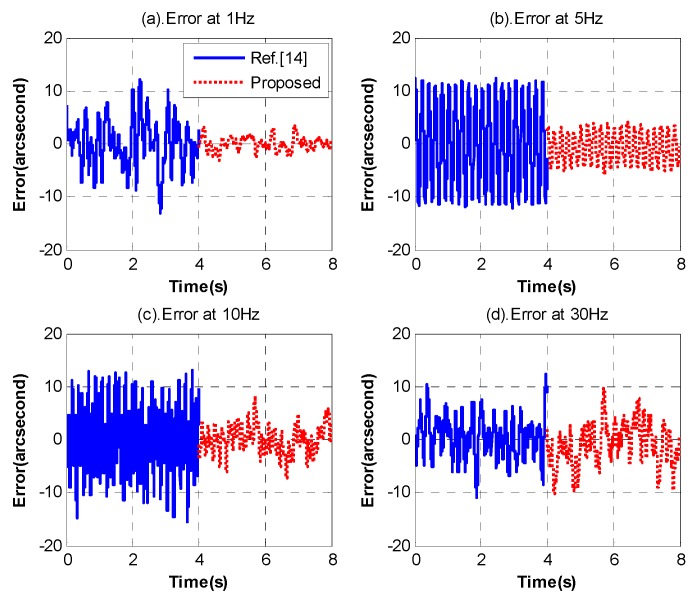
The residual stabilization errors in different frequencies.

Figure 14 presents the DS’s comparison of the proposed method and Reference [14]. Figure 15 displays the residual stabilization errors of the two methods in different frequencies. Obviously, whether in frequency domain or time domain, the promotion is very big and could reach 10 dB in low and medium frequency, which means we have succeeded in further enhancing the system’s DS. 

## 6. Conclusions

In the small and cost-efficient OCS platform, to save cost and space, we choose the MEMS accelerometer as the inertial stabilization sensor. At the same time, the proposed complimentary filter, with a small amount of calculation, saves expenses on computing devices. To fully release sensors’ potential and enhance the DS as much as possible, this paper attempted to fuse signals of the MEMS accelerometer and CCD twice to get two virtual sensors without extra investment. We improved the fusion method, rebuilt the acceleration model and proposed a new FDOB structure that was not dependent on the original object. What is more, we skillfully combined the disturbance feed forward control and the feedback control to increase the system’s robustness. Compared with the previous time-domain once fusion in Reference [14], the proposed frequency-domain fusion minimizes the effect of the accelerometers’ defect and the CCD’s time delay. These ideas are worth learning on other occasions. Experiments verified that the proposed method is valid.
